# Electrostatic Effects in the Folding of the SH3 Domain of the c-Src Tyrosine Kinase: pH-Dependence in 3D-Domain Swapping and Amyloid Formation

**DOI:** 10.1371/journal.pone.0113224

**Published:** 2014-12-09

**Authors:** Julio Bacarizo, Sergio Martinez-Rodriguez, Jose Manuel Martin-Garcia, Montserrat Andujar-Sanchez, Emilia Ortiz-Salmeron, Jose Luis Neira, Ana Camara-Artigas

**Affiliations:** 1 Department of Chemistry and Physics, Research Centre for Agricultural and Food Biotechnology (BITAL), University of Almería, Agrifood Campus of International Excellence (ceiA3), 04120, Almería, Spain; 2 Department of Physical Chemistry, Faculty of Sciences, University of Granada, 18071, Granada, Spain; 3 Department of Chemistry and Biochemistry, Arizona State University, Tempe, Arizona, 85287, United States of America; 4 Instituto de Biología Molecular y Celular, Universidad Miguel Hernández, 03202, Elche (Alicante), Spain; 5 Biocomputation and Complex Systems Physics Institute, 50009, Zaragoza, Spain; University of Pittsburgh School of Medicine, United States of America

## Abstract

The SH3 domain of the c-Src tyrosine kinase (c-Src-SH3) aggregates to form intertwined dimers and amyloid fibrils at mild acid pHs. In this work, we show that a single mutation of residue Gln128 of this SH3 domain has a significant effect on: (i) its thermal stability; and (ii) its propensity to form amyloid fibrils. The Gln128Glu mutant forms amyloid fibrils at neutral pH but not at mild acid pH, while Gln128Lys and Gln128Arg mutants do not form these aggregates under any of the conditions assayed. We have also solved the crystallographic structures of the wild-type (WT) and Gln128Glu, Gln128Lys and Gln128Arg mutants from crystals obtained at different pHs. At pH 5.0, crystals belong to the hexagonal space group P6_5_22 and the asymmetric unit is formed by one chain of the protomer of the c-Src-SH3 domain in an open conformation. At pH 7.0, crystals belong to the orthorhombic space group P2_1_2_1_2_1_, with two molecules at the asymmetric unit showing the characteristic fold of the SH3 domain. Analysis of these crystallographic structures shows that the residue at position 128 is connected to Glu106 at the diverging β-turn through a cluster of water molecules. Changes in this hydrogen-bond network lead to the displacement of the c-Src-SH3 distal loop, resulting also in conformational changes of Leu100 that might be related to the binding of proline rich motifs. Our findings show that electrostatic interactions and solvation of residues close to the folding nucleation site of the c-Src-SH3 domain might play an important role during the folding reaction and the amyloid fibril formation.

## Introduction

The non-receptor c-Src tyrosine kinase participates in different signaling processes by phosphorylation of tyrosine residues in a variety of proteins, modifying their properties, such as enzymatic activity, subcellular localization and stability. This protein has been implicated in the development, growth, progression and metastasis of several cancers [1]. The c-Src tyrosine kinase is made up of six functional regions: (i) a N-terminal fatty acid-acylation domain, (ii) a unique domain, (iii) a Src homology 3 (SH3) domain, (iv) a Src homology 2 (SH2) domain, (v) a catalytic domain, and (vi) a C-terminal regulatory domain. Of all those, the SH3 domain plays a key role in the regulation of the tyrosine kinase activity through the recognition of an intramolecular Proline Rich Motif (PRM) and, additionally, through the interaction with other PRMs present in other proteins.

The SH3 domain of the c-Src tyrosine kinase (c-Src-SH3) is one of the best-characterized SH3 domains, so far. The folding mechanism of the c-Src-SH3 domain has been extensively studied using both experimental and theoretical approaches [2]–[10]. Whereas the structure of this domain was first solved by NMR in 1992 [11], the first crystallographic structure was not described until 2009 [12]. The Gln128Arg mutant crystallizes as an intertwined dimer, where 3D-domain swapping takes place by exchange of the RT loop of two different protomers and the n-Src loop acts as hinge loop (see Results for a description of the structure of the protein).

To date, more than 50 proteins have been described to undergo 3D-domain swapping. However, the diversity of their sequences and secondary structures suggest that almost any protein may oligomerize via this mechanism [13]. In this way, the swapped-oligomers appear as an alternative folding of the protomer, which might be driven by: (i) a lower energy of the self-associated species, as compared with the single folded chain (thermodynamically favoured); or alternatively, (ii) the faster formation of the oligomer and its later stabilization (kinetically favoured). The interest in these oligomers resides in their plausible relationship with important diseases related to the amyloid formation [14] [15]. In this way, the formation of domain-swapped oligomers have been proposed as one possible mechanism underlying the appearance of early aggregates of amyloid fibril [16]. As a proof-of-concept, it has been described that some well characterized amyloid-forming globular proteins need to be unfolded through the formation of a domain-swapped species to develop amyloid aggregates [17]: prion [18], cystatin [19] and β-2-microglobulin [20]. Furthermore, oligomerization equilibria during the pre-nucleation phase of fibril assembly have also been described for the β-amyloid protein [21] and insulin [22].

To date, the formation of amyloid fibrils has been previously described in four SH3 domains: c-Yes [23], Fyn [24] (both from the family of the Src tyrosine kinases), PI3K [25] and the Asn47Ala mutant of α-spectrin [26]. Although oligomeric species have been previously described for the PI3K [27] and the mutant of α-spectrin [28] during the early stages of the fibril aggregation process, no intertwined structures have been reported yet for any of these four SH3 domains. However, there are other SH3 domains where the formation of domain-swapped dimers have been reported: Eps8 [29], p47phox [30], c-terminal SH3 domain of CRKL [31] and the c-Src-SH3 [12]. Particularly interesting is the case of the c-Src-SH3 domain, for which formation of domain-swapped structures and amyloid formation were previously predicted by Molecular Dynamics (MD) calculations [8].

Folding studies conducted on the c-Src-SH3 domain indicate that the diverging type II β-turn (residues 103–106) is able to fold independently [5]. At this loop, Glu106 side-chain forms a hydrogen bond with Ser123 side-chain at the distal loop (residues 123–127), and this interaction has been proposed to play a key role in the TS of the folding reaction acting as a nucleation site [32]. As for other SH3 domains, a two-state model has been proposed for the folding of c-Src-SH3 domain; however, the application of modern NMR relaxation dispersion techniques to the study of the folding of Fyn-SH3 domain has revealed the presence of intermediates along the folding pathway [33] [24]. These low populated intermediates cannot be observed using more traditional approaches for studying protein folding and may be common to the folding of other SH3 domains from the Src tyrosine kinase family.

To better understand the mechanism of 3D-domain swapping formation in c-Src-SH3 domain, and its plausible implication in amyloid fibril formation, we have solved the crystallographic structures of the WT and three mutants at position 128 of the c-Src-SH3 domain. This position is not conserved among the sequences of the c-Src-SH3, Yes-SH3 and Fyn-SH3 domains (being a glutamine, a lysine or a glutamic acid, respectively). The differences in charge of these residues might affect the electrostatic environment at this protein region, which has been proposed to be critical in the folding of the c-Src- and Fyn-SH3 domains [3]. Besides, this study pinpoints the importance of this position in the pH-dependent amyloid-formation of c-Src-SH3 domain.

## Materials and Methods

### Cloning, expression and purification of chicken c-Src-SH3 domain

The plasmid pET15b containing the chicken c-Src-SH3 domain gene was a generous gift from Dr. E. Freire (Johns Hopkins University, USA). The plasmid was expressed in *Escherichia coli* BL21 (DE3) strain (Novagen) with an N-terminal 6×-His-tag and an engineered thrombin cleavage site, which eliminates the histidine tag of the domain after purification whereas keeping the residues Gly81-Ser82-His83-Met84 as a cloning artefact. The Quikchange Site-Directed Mutagenesis Kit (Stratagene) was used for mutations. All the mutants at this work (Gln128Arg, Gln128Lys and Gln128Glu) were created using the pET15b plasmid containing the chicken c-Src-SH3 domain gene as a template. The full sequence of the WT variant is shown at S1 Figure. The protein was purified by a standard protocol [12], [34]. Protein purity and concentration were determined as described previously [12].

### Protein crystallization

Crystals of the c-Src-SH3 domain were obtained by vapour diffusion technique using a sitting or hanging drop setup at 25°C. The protein solution 10–15 mg·ml^−1^ (1.45–2.18 mM) in 10 mM Tris buffer at pH 8.0 was mixed with an equal volume of the reservoir solution and vapour equilibrated against 0.5–1 ml of the same solution. Crystals of the intertwined form of the WT and Gln128Arg/Lys/Glu mutants were obtained using a solution of 1.6–1.8 M ammonium sulphate, 10% PEG300, 10% glycerol and 0.1 M sodium acetate at pH 5.0. Crystals of the monomeric form of the WT and Gln128Glu mutant were obtained in 1.4–1.8 M ammonium sulphate, 5 mM NiCl_2_, 5 mM MBCD, 10% Glycerol, 0.1 M Hepes at pH 7.5. Micro-seeding techniques and addition of 10 mM NDSB-201 were used to improve the size and quality of the Gln128Glu crystals. Detailed crystallization conditions for each protein are given at Table 1.

**Table 1:** X-ray data collection and refinement statistics.

	WT MONOMER	WT DIMER	Q128E MONOMER	Q128E DIMER	Q128K DIMER	Q128R DIMER
PDB code	4JZ4	4JZ3	4OMO	4OMN	4OMP	4OML
Crystallization conditions	1.8 M (NH_4_)_2_SO_4_, 10% glycerol, 5 mM NiCl_2_, 0.1 M Hepes, pH 7.5	1.6 M (NH_4_)_2_SO_4_, 10% PEG 300, 10% glycerol, 0.1 M sodium acetate, pH 5.0	2M (NH_4_)_2_SO_4_,5 mM NiCl_2_, 5 mM MBCD, 0.1M Hepes, pH 7.5	1.8 M (NH_4_)_2_SO_4_, 10% PEG 200, 10% glycerol, 10 mM NDSB, 0.1M sodium acetate, pH 5.0	1.8 M (NH_4_)_2_SO_4_, 5%PEG 300, 10% glycerol, 0.1M sodium acetate, pH 5.0	1.6 M (NH_4_)_2_SO_4_, 10% PEG 300, 10% glycerol, 0.1M sodium acetate, pH 5.0
Data collection						
Wavelength (Å)	0.9334	0.9762	1.033	0.9795	0.9795	0.9775
Resolution (Å)	35.73-1.56 (1.61-1.56)	19.35 -1.85 (1.92-1.85)	45.46-1.04 (1.07 -1.04)	38.55-1.5 (1.53-1.50)	29.39-2.00 (2.06-2.00)	19.22-1.60 (1.63-1.60)
Space group	P2_1_2_1_2_1_	P6_5_22	P2_1_2_1_2_1_	P6_5_22	P6_5_22	P6_5_22
Unit cell (Å)	43.67 45.36 58.02	46.93 46.93 126.61	43.71 45.46 57.81	46.69 46.69 127.68	46.86 46.86 127.85	46.55 46.55 128.03
Total reflections	57432 (1795)	100654 (6547)	684773 (25814)	169041 (7821)	70435 (4940)	143425 (6524)
Unique reflections	16919 (813)	7649 (471)	56398 (2482)	14008 (651)	6002 (430)	11086 (503)
Multiplicity	3.4 (2.2)	13.2 (13.9)	12.2 (10.4)	12.1 (12.0)	11.7 (11.5)	12.9 (13.0)
Completeness (%)	99.6 (96.7)	99.8 (100.0)	99.2 (84.5)	99.9 (98.9)	98.6 (96.3)	97.0 (93.9)
I/σI	16.0 (2.0)	33.8 (5.6)	20.3 (3.4)	26.9 (4.6)	25.9 (5.0)	21.4 (4.4)
Wilson B-factor (Å^2^)	11.6	24.0	10.6	17.1	37.0	21.6
R-merge (%)	4.4 (34.0)	5.1 (58.1)	6.5 (74.5)	5.2 (68.6)	5.5 (51.6)	7.1 (6342)
Refinement						
R-work (%)	15.1 (23.1)	22.0 (27.3)	14.8 (23.5)	15.6 (18.7)	23.6 (30.1)	16.8 (20.2)
R-free (%)	17.6 (21.6)	24.10 (26.3)	16.8 (25.2)	16.8 (24.0)	26.1 (38.0)	19.9 (22.8)
Protein residues	118	57	120	57	56	57
Water	149	36	155	30	7	22
RMS bonds (Å)	0.010	0.006	0.011	0.009	0.009	0.012
RMS angles (degree)	1.202	1.046	1.41	0.97	1.15	1.23
Ramachandran favoured (%)	98	100	98	100	98	100
Ramachandran outliers (%)	1.8	0	1.7	0	0	0
Average B-factor (Å^2^)	18.8	35.8	16.7	29.9	46.5	37.9

Statistics for the highest-resolution shell are shown in parentheses.

### Data collection and refinement

Crystals were transferred into a cryoprotectant solution containing the reservoir solution before flash-cooling in liquid nitrogen. Data sets were collected at −173°C on the ID14-4 beamline at the ESRF (Grenoble, France) and Xaloc beamline at ALBA (Barcelona, Spain). Data were indexed and integrated with the program XDS [35], [36]. Data scaling was performed using the program Aimless [37] from the CCP4 suite [38]. The crystallographic parameters and statistics of data collection are listed in Table 1.

Solution and refinement of the structures were performed using the PHENIX suite [39]. Molecular-replacement phasing using PHASER [40] was performed with the coordinates of the crystallographic structure of the c-Src-SH3 Thr98Glu mutant complexed with the high affinity peptide APP12 (PDB code 4HVV) [41]. The water molecules, the peptide ligand and residues of the n-Src loop were removed from the model. Manual model-building was performed using COOT [42], [43]. Refinement was performed using phenix.refine in PHENIX [44]. In those datasets with resolution equal or higher than 1.6 Å, the final cycles of refinement were performed using anisotropic B-factors. Quality of the structure was checked using MOLPROBITY [45] and PROCHECK [46]. Refinement statistics are collected in Table 1.

Overall structure comparison and analysis were performed with the Structure Comparison GUI in Phenix Suite [39]. Structure superposition and RMSD calculations were performed using the CCP4 module LSQKAB [47]. Protein interfaces in the crystals were characterized using the PISA server [48]. Distances between amino acids and accessible surface area (ASA) were calculated using the program CONTACT and AREAIMOL from CCP4 Suite [38], respectively. Analysis of aggregation-prone regions were performed with the TANGO [49] and WALTZ [50] algorithm.

### Dynamic Light Scattering (DLS)

DLS experiments were performed in a Zetasizer nano instrument (Malvern Instrument Ltd, United Kingdom) equipped with a 10-mW helium-neon laser (λ = 632.8 nm) and a thermoelectric temperature controller. Experiments were analyzed with Zetasizer software (Malvern Instrument Ltd, United Kingdom). The hydrodynamic radius (*R*_h_) was determined as described elsewhere [41].

### NMR spectroscopy

The NMR data were acquired at 25°C on a Bruker Avance DRX-500 spectrometer (Bruker GmbH, Germany), equipped with a triple resonance probe and z-pulse field gradients. Processing of spectra was carried out with the XWINNMR software. Translational self-diffusion measurements (DOSY) were performed with the pulsed-gradient spin-echo sequence and dioxane was used as an internal references (with a hydrodynamic radius of 2.12 Å) [51]; data was analyzed as described elsewhere [52]. Sample concentration for the DOSY experiments was 10 mg·ml^−1^.

### Thermal denaturation

Thermal-denaturations were monitored by the intrinsic fluorescence of tryptophan and tyrosine residues on a Varian-Cary Eclipse Fluorescence Spectrophotometer (Agilent, USA) with excitation and emission slits of 5 nm. Excitation filter was fixed in automatic position and the emission filter was left open. Sample concentrations were 0.013 mg·ml^−1^ (2 µM) in 0.1 M buffer (sodium acetate pH 5.0, MES pH 6.0 and Hepes pH 7.0). All the measurements were performed using a 10-mm path length cell and changes at 315, 330 and 350 nm were monitored after excitation at 280 or 295 nm. The scan rate was 60°C·h^−1^, with an average time of 1 s, and data were collected every 0.2°C. The unfolding curves were analyzed using a two-state model as described previously [52]. The reported thermal denaturation midpoints for the proteins are the average of the three denaturations over the different wavelengths explored (Table 2).

**Table 2:** Thermal stability of WT and mutants of the c-Src-SH3 domain.

pH	Thermal denaturation temperatures (*T_m_*, °C)^a^			
	WT	Gln128Glu	Gln128Arg	Gln128Lys
**5.0**	76.3±0.3	71.2±0.2	80.8±0.6	79.7±0.3
**6.0**	76.8±0.4	72.4±0.4	80.8±0.4	81.8±0.4
**7.0**	76.7±0.4	71.6±0.5	83.1±0.5	82.0±0.2

a Errors are fitting errors to the two-state denaturation model.

### Thioflavin T Fluorescence experiments

Fluorescence spectra were recorded at 25°C in a FP-6500 spectrofluorimeter (Jasco Inc., Japan) equipped with an ETC 273T Peltier accessory. A 250 µM stock solution of ThT was prepared in 0.1 M buffer (sodium acetate and Hepes at pH 5.0 and 7.0, respectively). For each measurement, 10 µL of protein solution were mixed with 50 µL of ThT stock solution and adjusted to a final volume of 1 ml of 0.1 M of the corresponding buffer. Samples were subsequently incubated for one hour with agitation prior measurement; sample preparation was carried out in darkness to avoid dye decomposition. The sample was excited at 440 nm with a 10 nm slit width and the fluorescence emission spectra were recorded between 450 and 600 nm with a 5 nm slit width. All measurements were performed using a 10-mm path length cell with scanning speed of 500 nm·min^−1^. Protein concentration was 0.06 mg·ml^−1^ (10 µM).

### Congo red absorbance experiments

Congo Red (CR) experiments were carried out in a spectrometer PerkinElmer Lambda 25 (UV/VIS) at 25°C. A 20 mM CR stock solution was prepared in 0.1 M buffer (sodium acetate and Hepes at pH 5.0 and 7.0, respectively). For each measurement, 10 µL of protein solution were mixed with 20 µL of CR stock solution and adjusted to a final volume of 1 ml of 0.1 M of the corresponding buffer. The spectrum of each sample was recorded between 400 and 600 nm; the solution of the buffer was used as a blank. Protein concentration was 0.06 mg·ml^−1^ (10 µM).

### ANS binding assay

Fluorescence spectra were recorded at 25°C in a FP-6500 spectrofluorimeter (Jasco Inc., Japan) equipped with an ETC 273T Peltier accessory. A 0.1 mM ANS stock solution was prepared in 0.1 M buffer (sodium acetate and Hepes at pH 5.0 and 7.0, respectively). For each measurement, 10 µL of protein solution were mixed with 25 µL of ANS stock solution and adjusted to a final volume of 1 ml of 0.1 M of the corresponding buffer. Sample preparation was carried out in darkness to avoid dye decomposition. The sample was excited at 370 nm and the emission fluorescence spectra were recorded between 400 and 600 nm. All measurements were performed using a 10-mm path length cell with scanning speed of 500 nm·min^−1^ and excitation and emission slit widths of 5 nm. Protein concentration was 0.06 mg· ml^−1^ (10 µM).

### Transmission electron microscopy (TEM)

c-Src-SH3 domain samples were prepared at 25 mg·ml^−1^ (3.62 mM) in 0.1 M sodium acetate (pH 5.0) and incubated for several weeks at 25 and 37°C. Before measurements, 20 µL of protein sample were placed on a formvar-carbon-coated copper grid and left for 4 min at 25°C for sample adsorption. The grid was washed twice with double distilled water and the samples stained with 1% (w/v) uranyl acetate for 1 min. The dried samples were observed in an electron microscope operating at an accelerating voltage of 200 and 80 kV, Philips CM20 TEM-STEM (Germany) and Zeiss 902 (Switzerland) microscope, respectively (CIC services of the University of Granada, Spain).

## Results

### Crystal structures of monomeric WT and Gln128Glu mutant c-Src-SH3 domain

**Figure 1:**
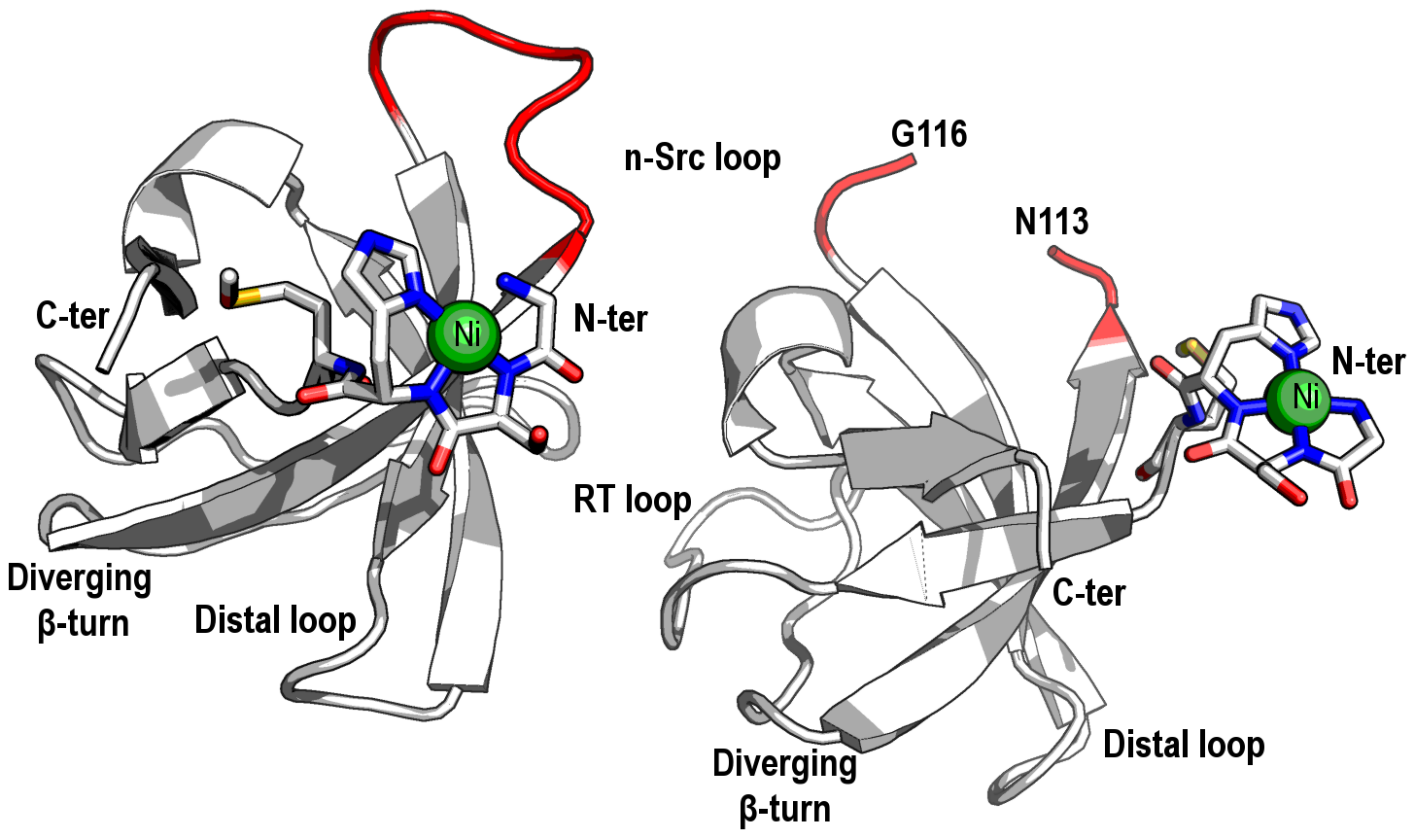
Overall fold of the monomeric structure of the c-Src-SH3 domain. Overall fold of the monomeric species of the WT c-Src-SH3 domain (WT_M_, PDB code 4JZ4). The AU is composed by two chains of the SH3 domain; both chains are represented as a cartoon (white). The n-Src loop residues in chains A and B are shown in red. In chain B, the poor electronic density in the difference maps does not allow to model residues 114-115. Both chains show a nickel-binding site at the N-terminal formed by the residues His83-Ser82-Gly81, with slight differences in the conformation and in the axial ligand (nickel ion is represented with a green sphere). All the figures were performed using the program Pymol 1.7 (distributed by Schrödinger).

The structures of the monomeric form of the WT (WT_M_) and Gln128Glu mutant (Q128E_M_) of the c-Src-SH3 domain were solved from crystals belonging to space group P2_1_2_1_2_1_ with two molecules in the AU. In each crystal structure, both molecules at the AU show the typical SH3 domain fold composed by five β-strands forming two tightly packed antiparallel β-sheets (Fig. 1): β1-strand (Phe86-Ala88); β2-strand (Arg107-Asn112); β3-strand (Trp118-Ser123); β4-strand (Thr129-Pro133); β5-strand (Val137-Pro139). The loops connecting these strands are referred to as the RT loop (Thr96-Asp99), the n-Src loop (Asn113-Gly116) and the distal loop (Ser123-Gly127); strands β4–β5 are separated by a 3_10_-helix (Ser134-Tyr136). Finally, a type II β-turn highly conserved among the SH3 domains [53], is formed by residues Lys103-Glu106. This turn is referred as *diverging* because of the lack of backbone-backbone hydrogen-bonds between the two connecting strands. In both structures, whereas chain A could be completely modeled (amino acids 81-140), some residues at the n-Src loop of chain B could not be modeled because of the lack of electron-density in the difference maps. Analyses of both monomeric structures have shown that all the residues present ψ and φ angles within allowed regions of the Ramachandran plot, aside from residue Ser82. This residue belongs to the cloning tail (residues Gly81-Ser82-His83-Met84) generated from the thrombin cleavage of the His-tag cloned protein. Residues belonging to this cloning tail participate in the binding of a Ni^2+^ ion through their backbone nitrogen atoms and the N^δ1^ atom of His83 side-chain (Fig. 1).

**Figure 2:**
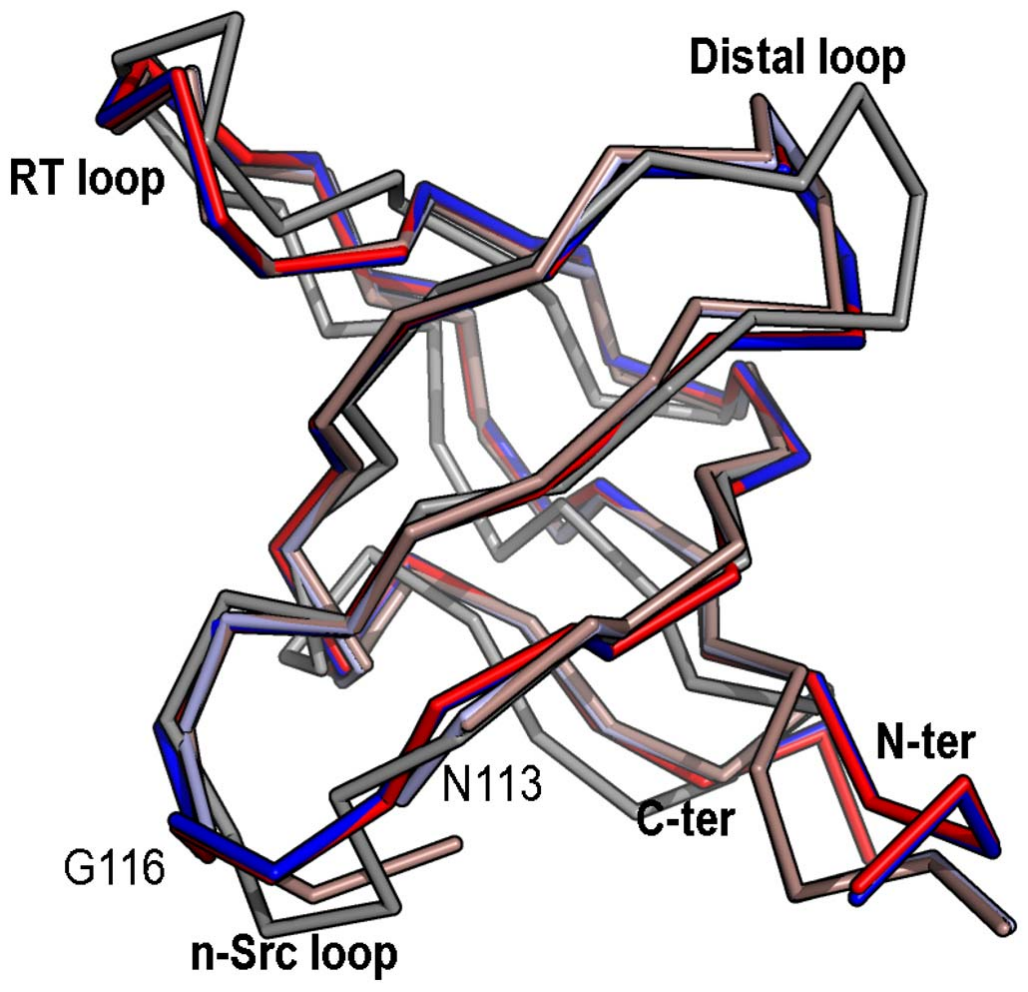
Superposition of the monomeric structures of the c-Src-SH3 domain. Superposition of the crystallographic structures of the monomeric c-Src-SH3 domain (WT_M_, chain A blue and B clear blue, PDB code 4JZ4; Q128E_M_, chain A red and B clear red, PDB code 4OMO) with that solved by NMR (PDB code 1SRL) (grey).

**Figure 3:**
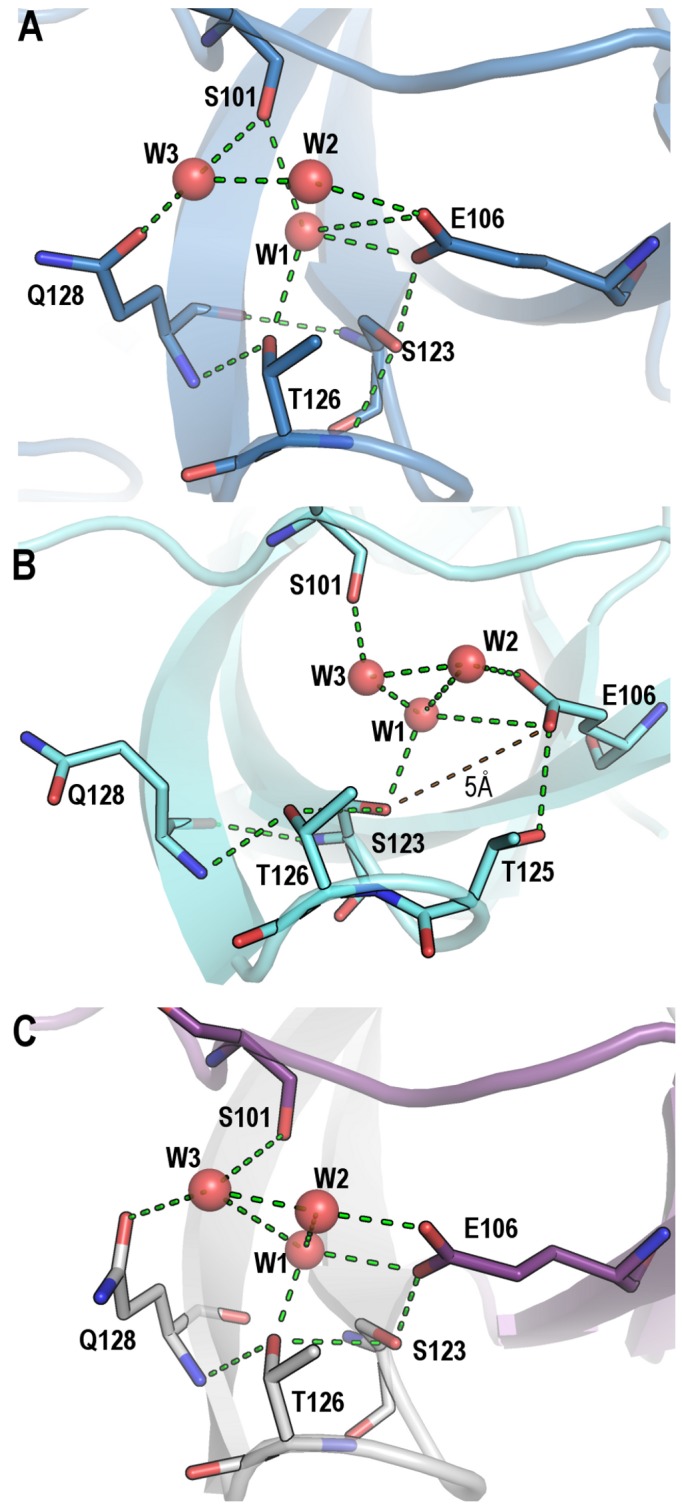
Nucleation site of the WT c-Src SH3 domain. Hydrogen-bond interactions among the residues belonging to the diverging β-turn and those of the distal loop are shown in green dotted lines. WT_M_ (PDB code 4JZ4) chains A (panel A) and B (panel B) are shown in blue and cyan, respectively. (C) Intertwined dimer structure of the WT c-Src SH3 domain (PDB code 4JZ3), residues at chain A are shown in white sticks and those belonging to the symmetry related molecule (chain B) are in magenta sticks.

For comparison of the two monomeric crystal structures superposition of chains A of the WT_M_ and Q128E_M_ structures were carried out, resulting in a RMSD value of only 0.15 Å. The same result was obtained for chains B, with a RMSD value of 0.12 Å. However, comparison of the two chains (chain A and B) belonging to the WT_M_ showed larger differences between them. The same result was obtained for the Q128E_M_. In both structures, the main differences between chains A and B are located at the distal and n-Src loops (Table 3). Those differences are even higher when comparing these structures with the average WT NMR structure (PDB code 1SRL) [54] (Fig. 2). Conformational changes at the n-Src loop are important because the specificity in the binding of PRMs to SH3 domains has been attributed to the interaction of the residues flanking the canonical binding motif (P*xx*P) with some residues at the RT loop and n-Src loop [55]. In addition, the distal and diverging β-turn loops play a relevant role in the folding reaction of the c-Src-SH3, and are considered as the folding nucleation site: both loops are formed early in the folding reaction and are brought together by the hydrogen-bond formed by the side-chains of Glu106 and Ser123 [3]. The conformational changes observed at the distal loop between the two chains in the AU in both structures seem to be related to the arrangement of the hydrogen-bond network around Glu106, at the diverging β-turn, and several residues at the distal loop (Table 4). One of the advantages of the availability of the first X-ray structure of the monomeric c-Src-SH3 domain is the possibility to analyze the solvent molecules, not observed in previous NMR structures. In both monomeric structures, the side-chain of residue Glu106 in chain A (Glu106_A_) is hydrogen-bonded to Ser123_A_ side-chain and two water molecules, thereafter W1 and W2. A third water molecule, W3, is placed at hydrogen-bond distance of Gln128_A_ side-chain (or Glu128_A_ in the Gln128Glu mutant), the carbonyl atom of Ser101_A_ and W2 (Fig. 3A). However, in chain B, Glu106_B_ side-chain is hydrogen-bonded to Thr125_B_ side-chain (2.8 Å) instead of Ser123_B_ side-chain (distance between Glu106_B_ and Ser123_B_ is 5 Å). W1 and W2 molecules linked to Glu106_B_ side-chain are also maintained, but not W3: the latter is present in the WT structure, but not in the mutated one. Gln128_B_ (or Glu128_B_) side-chain is oriented outwards from the pocket formed between the diverging β-turn and the distal loop, and therefore, its interaction with W3 is lost (Fig. 3B).

**Table 3:** Comparisons in terms of RMSD (Å).

Reference Structure	Superpose Structure	All atoms^a^ (Å)	Cα (Å)
**WT_M_ chain A**	WT_M_ chain B	1.42	0.83
	NMR structure (1SRL)	2.56	1.82
	Q128E_M_ chain A	0.60	0.15
	Q128E_M_ chain B	1.33	0.84
	WT_D_ N-terminal^b^	1.93	0.76
	WT_D_ C-terminal^c^	0.93	0.26
**WT_M_ chain B**	NMR structure (1SRL)	2.09	1.45
	Q128E_M_ chain A	1.33	0.84
	Q128E_M_ chain B	0.50	0.12
	WT_D_ N-terminal^b^	1.55	0.59
	WT_D_ C-terminal^c^	1.07	0.42
**WT_D_**	Q128E_D_	0.74	0.36
	Q128K_D_	0.94	0.31
	Q128R_D_	0.73	0.26
**Q128E_M_ chain A**	Q128E_M_ chain B	1.96	1.23

a Alignment performed taking into account only non-hydrogen atoms.

b Alignment performed taking as reference residues 84-110 of the monomer.

c Alignment performed taking as reference residues 117-140 of the monomer.

**Table 4:** Hydrogen-bond distances in the water network at the distal loop and diverging β-turn.

	W1 Distance (Å) WT_M_/Q128E_M_		W2 Distance (Å) WT_M_/Q128E_M_		W3 Distance (Å) WT_M_/Q128E_M_	
	Chain A	Chain B	Chain A	Chain B	Chain A	Chain B
**Ser101 (O)**	2.82/2.86	3.53/3.49			3.07/3.04	2.51
**Glu106 (OE1)**	3.54/3.62	2.71/2.76	2.75/2.75	3.25/3.34		
**Glu106 (OE2)**	2.69/2.73	3.62/3.68		2.83/2.72		
**Ser123 (OG)**	3.67/3.68	2.67/2.65				
**Thr126 (OG1)**	2.72/2.71					
**Gln128(OE1)**			2.99/-		2.62/-	
**Gln128(NE2)**					3.45/-	
**Glu128(OE1)**					2.74/-	
**Glu128(OE2)**					3.71/-	
**W1**			3.77/3.77	3.48/3.47	3.66/3.67	2.47/-
**W2**	3.77/3.77	3.48/3.47			2.69/2.75	2.87/-
**W3**	3.66/3.67	2.47/-	2.69/2.75	2.87/2.73		
**W4**						2.60/-

**Figure 4:**
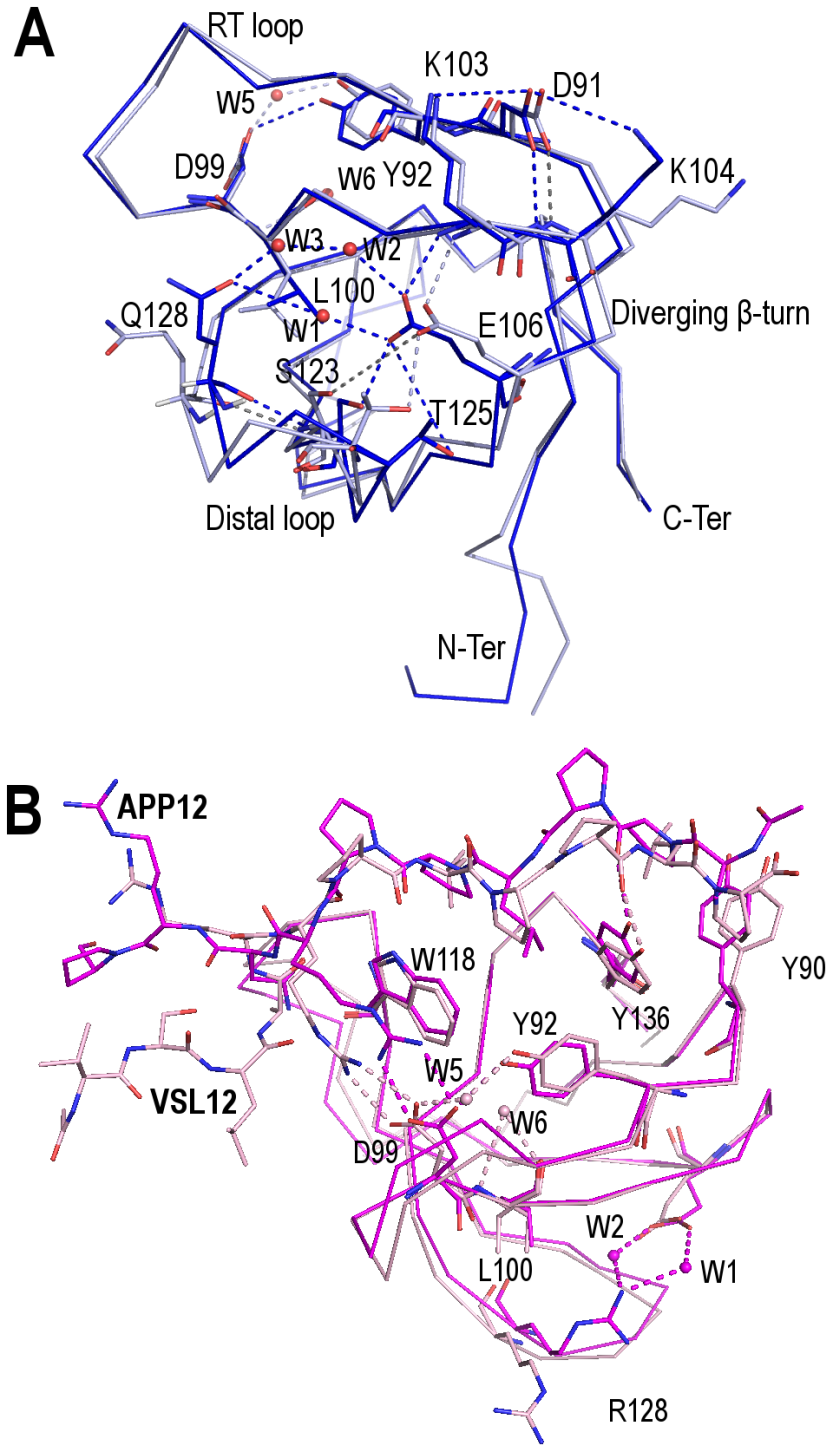
Conformational changes in the c-Src-SH3 domain. Conformational changes at the distal loop of the WT c-Src-SH3 domain (PDB code 4JZ4). Chains A (blue) and B (light blue) main-chain are shown in ribbon, relevant residues are represented with sticks and water molecules with spheres. The hydrogen-bond network is represented with dashed lines in the color of the corresponding chain. (A) Changes at Leu100 are correlated with a different hydrogen-bond network between the diverging β-turn and distal loop; this hydrogen-bond scaffold also affects to the PRMs binding site. (B) Comparison of the crystallographic structures of the APP12 (magenta) and VSL12 (light pink) complexes of the double Thr98Glu-Gln128Arg mutant (PDB codes 4HVV and 4HVW, respectively). The main-chain is represented in ribbon and relevant residues are represented in sticks. The hydrogen-bond network is shown in dashed lines with the color of the corresponding chain. Water molecules at the binding site of the c-Src-SH3/VSL12 complex have been represented with spheres (light pink) (W5 and W6). These water molecules are lost in the c-Src-SH3/APP12 complex where the relevant water molecules at the folding nucleation-site have been represented with spheres (magenta) (W1 and W2).

Changes at the folding nucleation site also affect residue Leu100, which is conserved among SH3 domains, and is part of the hydrophobic core with an ASA value <10 Å^2^. This residue shows different rotamers at each chain (Fig. 4A): rotamer *tp* in chain A vs rotamer *mt* in chain B. The presence of the rotamer *mt* is allowed by the displacement of the backbone of the distal loop (distance between A and B chain backbone atoms was ∼1.5 Å) and the low steric hindrance of the nearby residue, Gly130. In chain A, besides rotamer *tp* of Leu100_A_, we have also observed a hydrogen-bond between Asp99_A_-Tyr92_A_ side-chains. These residues are placed at the binding site and have implications in the binding specificity and affinity of PRMs: the formation of a salt bridge between Asp99 and some positively charged residues flanking the P*xx*P canonical binding motif drives the orientation of the PRMs [55]. In chain B, Asp99_B_ and Tyr92_B_ side-chains are ∼5 Å distance, but they are linked by a water molecule (W5) (Fig. 4A). The presence of the former water molecule and the slight displacement of the backbone of Leu100 and nearby residues (∼0.4 Å) generates a cavity that is filled by another water molecule (W6) which is fully buried (ASA 0 Å^2^). This water molecule is bound to the backbone nitrogen and oxygen atoms of Leu100_B_, and to the backbone oxygen of Tyr131. Interestingly, the same differences in: (i) the Leu100 rotamer; (ii) the hydrogen-bond between Asp99_A_-Tyr92_A_ side-chains; and (iii) the position of the water molecules has been found in the crystallographic structures of the complex with the high affinity synthetic peptides VSL12 (class I) and APP12 (class II) [41]. These differences are not attributable to differences in crystal packing between chains A and B at the AU as the structures of these complexes and the monomeric structures have been obtained from different crystals forms; furthermore, these conformational changes have also been observed in the NMR structures of the Fyn-SH3 domain [24]. While the conformation and hydrogen-bond between Asp99_A_-Tyr92_A_ present at chain A also appears in the crystallographic structure of the c-Src-SH3/APP12 complex (PDB code 4HVV), the Asp99_A_-W5-Tyr92_A_ arrangement found in chain B is present in the crystallographic structure of the c-Src-SH3/VSL12 complex. All these pieces of evidence indicate that Leu100 rotamer seems to be affected by the conformation of the distal loop; these changes seem to involve Asp99 and other residues that affect the binding affinity and specificity of PRMs. In these interactions, W6 can play a key role as it is hydrogen-bonded to Tyr131, which is also part of the specificity pocket of this SH3 domain. In our experience, since Leu100Ala mutation has been described to substantially reduce the affinity of the protein for the peptide substrates and its stability [5], [56], our results show that changes in the conformation of this residue might be correlated with the organization of the residues at the binding site to accommodate peptides of different nature (Fig. 4B).

### Crystal structures of the dimeric intertwined forms of WT and mutated c-Src-SH3 domain

**Figure 5:**
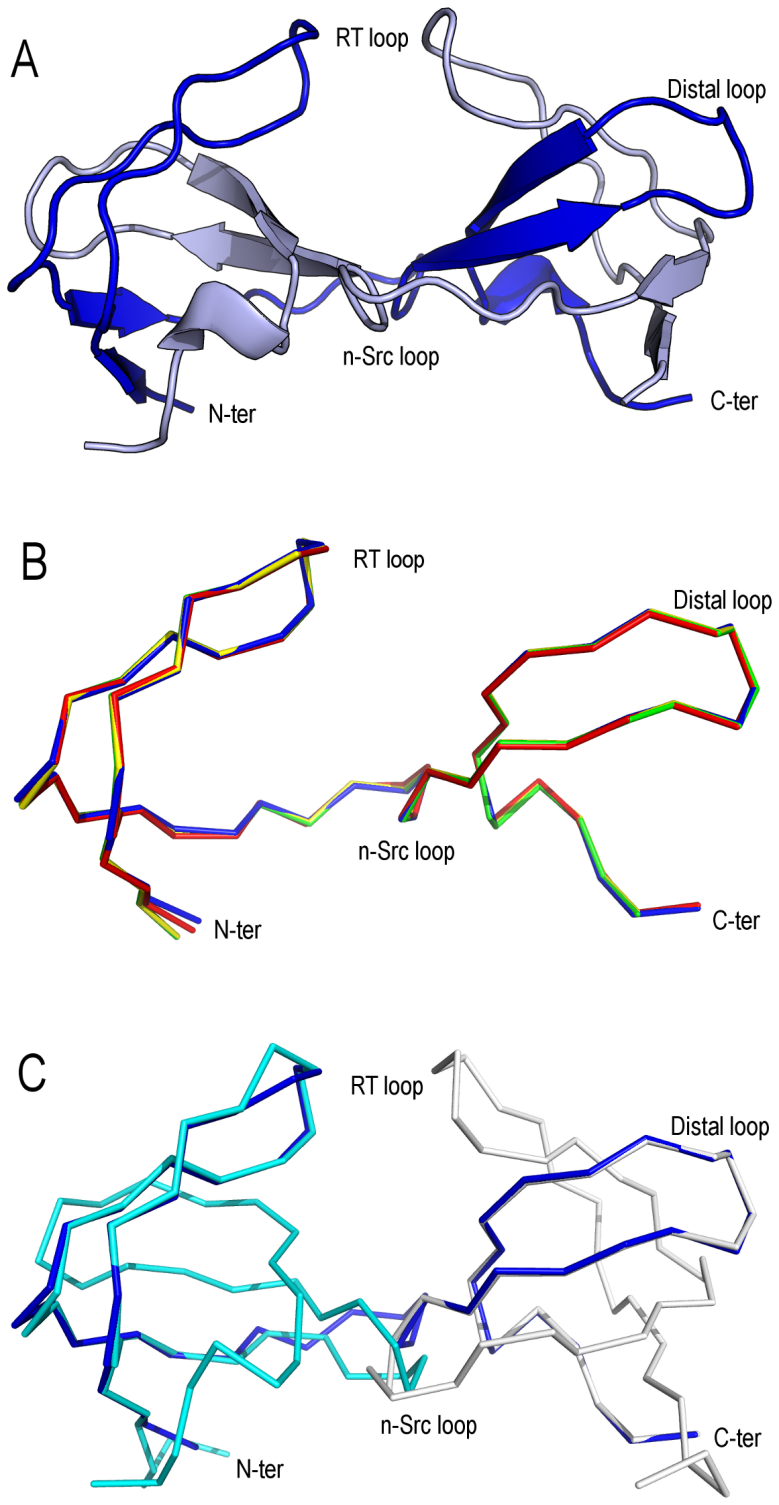
Intertwined dimer structures of the c-Src-SH3 domain. (A) Structure of the intertwined dimer of the c-Src-SH3 domain. Open chain of the WT c-Src-SH3 domain is shown in cartoon (blue). Chain B, which generates the dimer, was obtained by symmetry (light-blue). (B) Superposition of the intertwined structures of the WT_D_ (blue) and Q128E_D_ (red), Q128K_D_ (yellow), Q128R_D_ (green) mutants. (C) Superposition of the open chain of the intertwined dimer structure of the WT c-Src-SH3 domain (blue) (PDB code 4JZ3) to chain A of the monomeric structure of the WT (PDB code 4JZ4): the overlay of the N- and C-terminal regions is shown in cyan and white, respectively.

All the crystals of the dimeric form of the c-Src-SH3 domain belong to the hexagonal space group P6_5_22 and show a single chain of the c-Src-SH3 domain (Table 1). The intertwined dimer is generated by symmetry, and the structural motifs of the SH3 domain are formed by two polypeptide chains: residues (N-terminal)–111 from chain A and residues 117–(C-terminal) from the chain belonging to a symmetry mate (thereafter, chain B in the intertwined dimer structure). In all the intertwined structures, residues Met84 to Ser140 were modeled and all of them have φ and ψ angles values within the allowed regions of the Ramachandran plot. 3D-domain swapping of the polypeptide chains occurs as a result of an open-like conformation of the n-Src loop (Asn112-Gly116) which acts as a hinge loop (Fig. 5A). Superposition of the intertwined structures, using the WT_D_ as reference, resulted in RMSD values between 0.74–0.94 Å for all atoms and 0.26–0.36 Å for the Cα atoms (Fig. 5B) (Table 3). These low RMSD values indicate that these structures can be considered basically the same, except for some differences at the water hydrogen network around residue 128.

**Figure 6:**
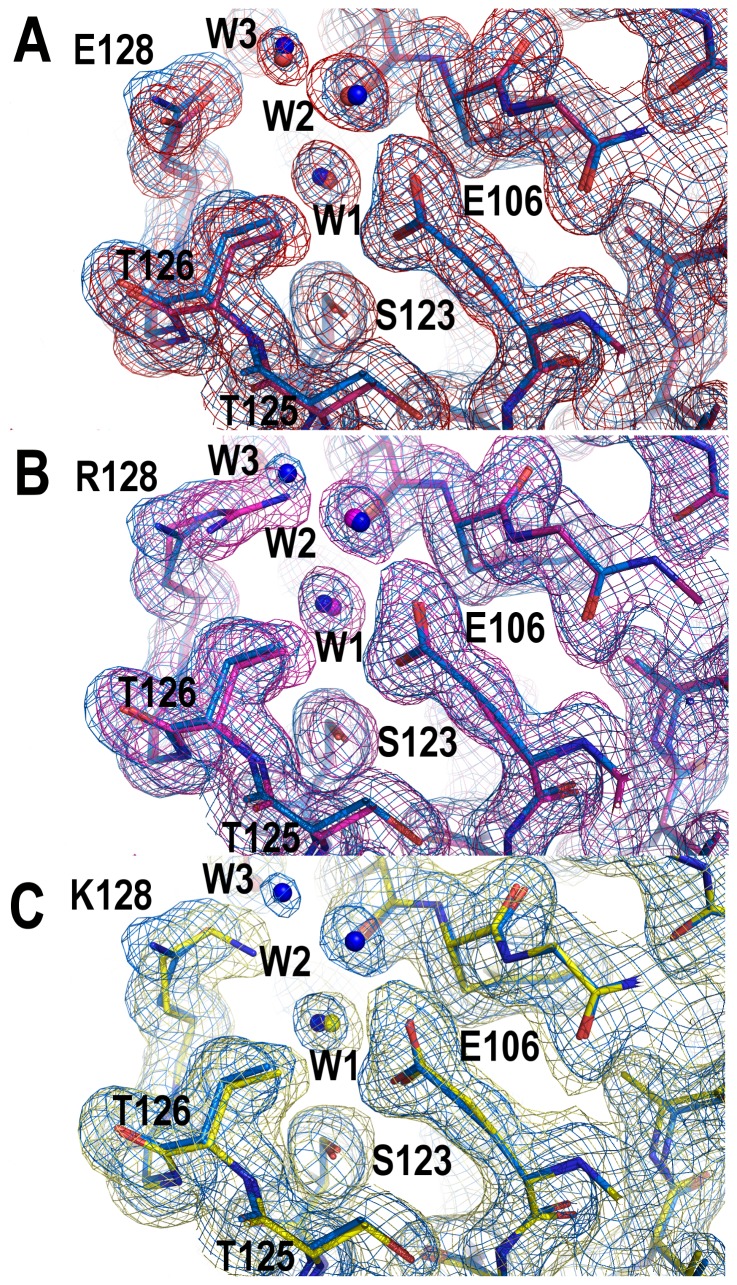
Water network at the nucleation site of the intertwined structures of the c-Src-SH3 mutants. Representation of the superposition of the 2Fo-Fc electron density map of the distal loop and the diverging β-turn of the WT (blue) and (A) Gln128Glu (red); (B) Gln128Arg (magenta); and (C) Gln128Lys (yellow) mutants. W1, W2 and W3 present in each structure are shown in the same color as the corresponding coordinates.

In this way, in the WT_D_, the same as in the WT_M_, three water molecules (W1, W2 and W3; see above) are hydrogen-bonded to residues in the distal loop and the diverging β-turn. The same arrangement is found in the Q128E_D_ (Fig. 6A). On the other hand, in the Q128R_D_, the longer side-chain of arginine displaces W3 (Fig. 6B), while W1 and W2 are placed at the same position; and in the Q129K_D_ the sole water molecule conserved is W1 (Fig. 6C), which is placed at hydrogen-bond distance of Lys128 and Glu106. Thus, the amino acid at position 128 is responsible for the hydrogen-bond scaffold around those water molecules. Interestingly, W1 is the only water molecule conserved among the different structures: it shows very low B-factors and small ASA values in all the structures in this work. These findings indicate that W1 is buried and tightly bound to the side-chain of Glu106, and should be considered a structural water molecule.

All the intertwined dimer structures described at this work show the presence of a PEG molecule at the interface of the two chains forming the dimer. This molecule crosses a two-fold crystallographic symmetry axis and the molecule of PEG modelled and its symmetry-related molecule are at the same position. To account for the special position, the PEG molecule has been modelled by giving half occupancy. The PEG location is symmetric, and hence this does not violate the crystal symmetry. As described before, this molecule participates in several hydrogen-bonds with residues at the dimer interface and water molecules and might have some role in the stabilization of the intertwined dimer (S2 Figure) [12].

### Comparison of the monomeric and dimeric structures

The comparison of the monomeric and dimeric structures of the c-Src-SH3 domain has been performed by superposition of the N-terminal (84-110) and C-terminal residues (117-140) of chain A of the WT_M_ to the corresponding residues in the open protomer of the intertwined dimers (Table 3). The RMSD values are low for most of the residues compared among the structures, with the exception of those present in the RT and distal loops (Fig. 5C).

The crystal interfaces of the monomeric and dimeric structures were compared using the PISA server [48]. In the swapped structures in this work, we can distinguish between: (i) the interactions that generate the closed-interface of the domain swapped dimer, which are already present in the monomer fold; and (ii) the interactions not present in the monomer, which form the new open-interface [57]. Analysis of the dimeric structure interfaces showed the largest differences in the hydrogen-bond pattern between the monomeric and dimeric structures to be located around the n-Src (hinge) loop (Asn112-Gly116). In the WT_D_, Glu115_A_ side-chain is hydrogen-bonded to Trp118_A_, Asn113_A_ and Tyr131_B_ side-chains (residue from chain B) and none of these interactions are present in the monomeric structures. Another hydrogen-bond present in the open-interface is formed between Asp117_A_ and Asp117_B_ side-chains (2.8 Å) where, as well, Trp119 side-chain participates (3.2 Å). This interaction is pH-dependent as it is only possible if the aspartate residue is protonated to avoid the repulsion of the negatively charged residues. PISA analysis also indicates the presence of a salt-bridge (namely, Glu115_A_-Arg95_B_ and Glu115_B_-Arg95_A_), which also belongs to the open interface and links the n-Src and RT-loops (Fig. 5B). These interactions can account for the differences in the RT-loop fold between the monomeric and dimeric structures of the c-Src-SH3 domain, and together with the Asp117_A_-Asp117_B_ hydrogen-bond, might contribute to the stabilization of the n-Src loop that it is fully-modeled in all the intertwined structures.

At the distal loop and diverging β-turn, the hydrogen-bond pattern of the dimeric structures resembles that found at chain A of WT_M_ and Q128E_M_: the residue at the position 128 is linked to Glu106 through the W1–W2–W3 water molecules network (Fig. 3C). Besides, in all the dimeric structures, the hydrogen-bond between Glu106_A_ and Ser123_B_ side-chains (or Glu106_B_ and Ser123_A_) is conserved with an average distance of 2.8 Å. Finally, the side-chain of the residue at position 128 is oriented to the pocket between the distal loop and the diverging β-turn. All the dimeric structures show: (i) the same Leu100 rotamer, *tp*, which is identical to that present in chain A of the monomeric structure (see above); and, (ii) the hydrogen-bonded Asp99-Tyr92 side-chains.

### Thermal stability of the Gln128 mutants

Since Gln128 seemed to be a key residue in dimer formation, we wanted to rule out whether it might have some effect on the stability of the protein. As dimer formation was shown to be pH-dependent, we carried out measurements at three different pH values (5.0, 6.0 and 7.0). The c-Src-SH3 domain has four tyrosine and two tryptophan residues, which allow stability to be monitored by fluorescence spectroscopy. Table 2 shows the dependence of the *T_m_* with the pH for the three mutants and WT c-Src-SH3 domain. Whereas the WT did not show a large pH-dependence of the *T_m_*, our results confirm that: (i) the mutation of Gln128 to arginine or lysine, which bears a positive charge, results in the stabilization of the c-Src-SH3 domain, since these species unfold at higher temperature than the WT (Table 2); and (ii) mutation to glutamate, which bears a negative charge, results in the destabilization of the c-Src-SH3 domain.

### Aggregation state of the c-Src-SH3 domain

In order to clarify the pH-dependence of the obtained crystal forms and their oligomerization state in solution, we carried out DLS and NMR experiments at two different pHs (5.0 and 7.0), in the presence and absence of 5% PEG300 at 25°C. We also studied the addition of PEG300, as the dimeric forms of the WT and the mutants showed the presence of a PEG molecule located at the swapped-dimer interface, which seems to favour the formation of the intertwined structure [12].

**Figure 7:**
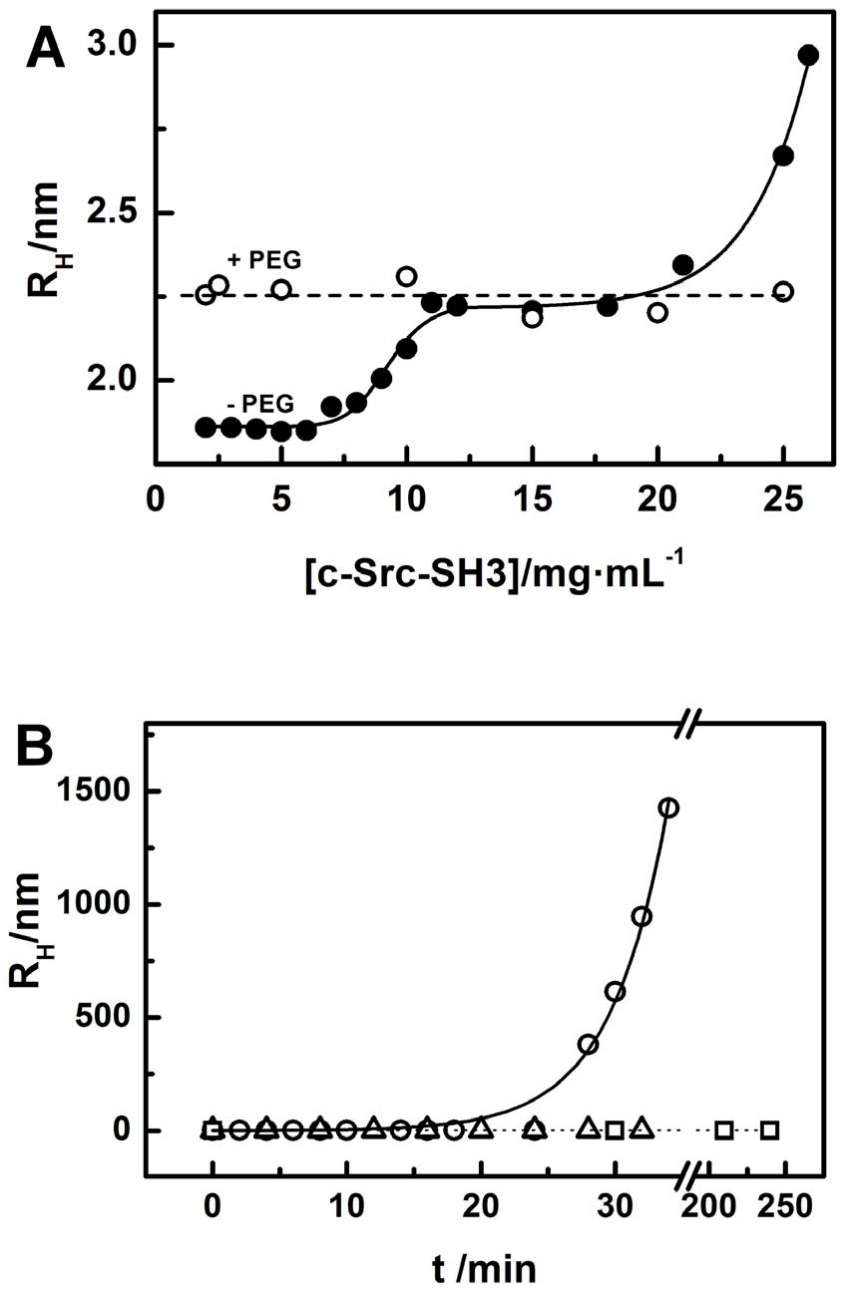
DLS experiments. (A) Average *R*_h_ as a function of c-Src-SH3 domain concentration, in 0.1 M sodium acetate (pH 5.0) at 25°C. Symbols represent measured data in the presence of 5% PEG300 (open circles) and absence of PEG300 (filled circles). (B) Aggregation kinetics of c-Src-SH3 followed by DLS. The protein at a concentration of 25 mg·ml^−1^ (3.6 mM) in 0.1 M sodium acetate pH 5.0 was incubated at 25°C (square) containing 5% PEG300 and without PEG300 at 20°C (triangles) and 25°C (circles) as a function of time.

DLS measurements indicate that at pH 7.0 the protein is a monomer in all the range of concentrations assayed, whether or not PEG is present. But at pH 5.0, changes in the value of the hydrodynamic radius (*R_h_*) were observed, indicating alterations in the aggregation state of the protein. Fig. 7A shows the dependency of the *R_h_ vs* protein concentration, in the absence and presence of low molecular-weight PEGs, at pH 5. The WT and mutated c-Src-SH3 domains, at protein concentrations lower than 5 mg·ml^−1^ (0.72 mM) and in the absence of PEG, were monomers with *R_h_* values of 1.8±0.1 nm. As the protein concentration raised, the *R_h_* value increased up to reach a value of 2.3±0.2 nm at protein concentrations of 15 mg·ml^−1^ (2.17 mM). Interestingly, the WT protein shows a noticeable change in the *R_h_* value at 25°C and at protein concentrations higher than 20 mg·ml^−1^ (2.90 mM), which indicates additional self-association happening within a few hours (Fig. 7B). However, at temperatures below 20°C, we did not observe the formation of high molecular-weight aggregates after several days. It is noteworthy that, under the same conditions assayed for the WT c-Src-SH3 domain, we did not detect any aggregation in the Gln128Arg/Lys mutants, even though these mutants were also dimers at protein concentrations higher than 5 mg·ml^−1^ (0.72 mM). Nevertheless, we detected high molecular-weight aggregates in the Gln128Glu mutant at pH 7.0 after long incubation times at 37°C. The addition of sodium chloride and an increase of the temperature accelerated this aggregation process.

In the WT and all the mutants, the addition of 5% PEG300 favours the formation of the dimer even at low protein concentrations. Furthermore, the presence of the crowding agent also stabilizes the dimer at high protein concentrations as can be concluded from the lack of formation of higher molecular-weight aggregates in the WT species (Fig. 7A); in addition, DLS experiments indicate that solutions of the WT and mutants of the c-Src-SH3 domain in presence of 5% PEG300 are monodisperse. The monomeric and dimeric states in solution of the WT were also assayed by DOSY-NMR. At mild acid pHs, WT c-Src-SH3 domain solutions (at 10 mg·ml^−1^ (1.4 mM)) showed a translational diffusion coefficient (*D*) of (1.04±0.01)·10^−6^ cm^2^·s^−1^, which results in a *R*_h_ of 1.5±0.2 nm, suggesting the presence of a monomer in solution. The addition of 5% PEG300 resulted in a decrease of the diffusion coefficient, (8.7±0.3)·10^−7^ cm^2^·s^−1^, which corresponds to the dimer, at pH 5.0 (the calculated *R*_h_ is 1.8±0.3 nm). However, at pH 7.0 a value of (1.13±0.02)·10^−6^ cm^2^·s^−1^ in the presence of PEG, and (1.12±0.02)·10^−6^ cm^2^·s^−1^ in the absence of PEG, obtained, in agreement with the presence of a monomer in solution.

The *R_h_* value was also calculated from the X-ray models using the HYDROPRO software [58]. The theoretical *R_h_* values of the WT_M_ and WT_D_ models were 1.63 and 2.03 nm, respectively, very close to the experimental values obtained by DLS experiments, and similar to those from NMR.

These results in solution might explain why at mild acid pHs the protein crystallizes as an intertwined-dimer and the difficulties to crystallize the protein in absence of low molecular weight PEGs.

### Amyloid formation in the c-Src-SH3 domain

**Figure 8:**
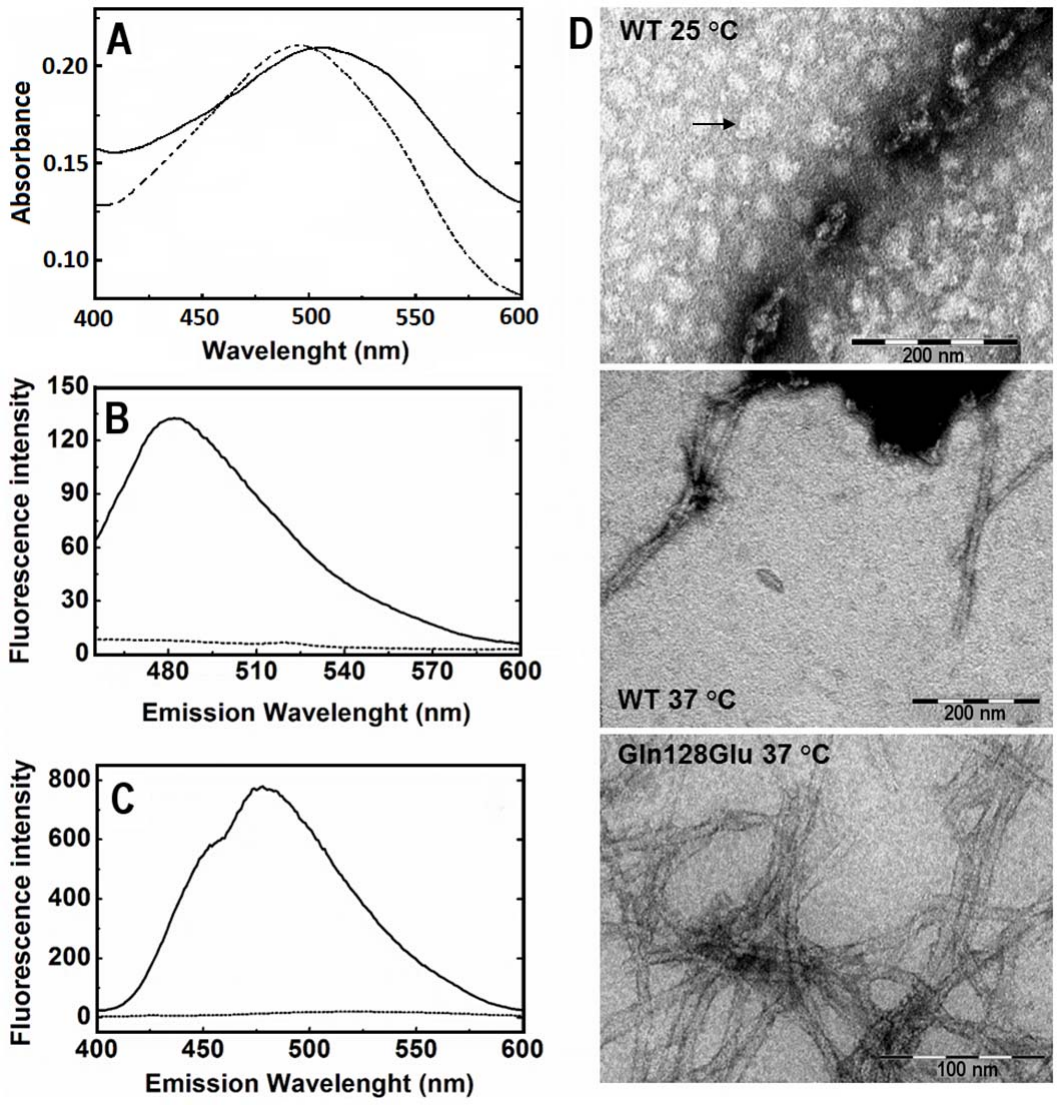
Characterization of amyloid aggregates of the c-Src-SH3 domain. WT c-Src-SH3 amyloid samples were prepared by incubation for one hour at 25°C of a protein solution at 25 mg·ml^−1^ (3.6 mM) in 0.1 M sodium acetate pH 5.0. A fresh WT sample solution at 10 mg·ml^−1^ (1.4 mM) in 0.1 M Hepes (pH 7.0) was used as control. After dilution in the appropriate buffer containing the dye solution to yield a final protein concentration of 0.06 mg·ml^−1^ (10 µM), the spectra were recorded. (A) Absorbance spectrum of 20 µM CR of the native protein (dotted line) and the presence of amyloid aggregates (continuous line). (B) Fluorescence emission spectra of 12.5 µM ThT solution of the native protein (dotted line) and amyloid aggregates (continuous line). (C) Fluorescence emission spectra of 25 µM ANS solution of the native protein (dotted line) and amyloid aggregates (continuous line). Similar spectra were recorded using the Gln128Glu mutant amyloid samples obtained by incubation for two weeks at 37°C of a protein solution at 1.4 mM (10 mg·ml^−1^) in 0.1 M sodium chloride, 0.1 M Hepes pH 7.0. (D) Negatively stained TEM images illustrating the diverse morphology of the aggregates formed by the c-Src-SH3: WT incubated for two weeks in 0.1 M sodium acetate (pH 5.0) at 25°C shows the presence of amyloid oligomers (one of them is indicated with a black arrow). Images of the WT incubated for two weeks in 0.1 M sodium acetate (pH 5.0) at 37°C show the presence of amyloid fibrils; and Gln128Glu mutant incubated for two weeks in 0.1 M Hepes 0.1 M sodium chloride (pH 7.0) at 37°C, which also shows the presence of long amyloid fibrils.

DLS experiments carried out with the WT at pH 5.0 at protein concentrations higher than 20 mg·ml^−1^ (2.90 mM), point out the formation of high molecular-weight aggregates in mild acid solutions at temperatures higher than 25°C. After one hour of incubation, the resulting solution had a gel-like appearance. This behavior was also observed in the Gln128Glu mutant, but in the latter the aggregates only appeared at neutral pH and after two weeks of incubation at 37°C. The presence of amyloid fibrils in these solutions was further characterized by dye-staining, such as CR, ThT and ANS as described elsewhere in the literature [59]. The CR UV-Vis spectrum showed the characteristic red shift of amyloid aggregates (Fig. 8A). In addition, ThT and ANS binding assays experiments (Figs. 8B and C) showed a notable increase of the fluorescence emission, which is also indicative of amyloid formation.

Finally, we verified amyloid fibril formation by transmission electron microscopy (TEM) (Fig. 8D). Amyloid solution of WT were prepared by incubation of 25 mg·ml^−1^ (3.6 mM) protein solution in 0.1 M sodium acetate buffer (pH 5.0) at 25 and 37°C for two weeks. The Gln128Glu amyloid solution was obtained by incubation of a 10 mg·ml^−1^ (1.4 mM) protein solution in 0.1 M Hepes (pH 7.0) at 37°C for two weeks in the presence of 0.1 M of NaCl. In the WT, amyloid oligomers were observed by TEM after two weeks of incubation at 25°C. These oligomers might be on a productive pathway for the formation of amyloid fibrils as it has been found in the amyloid-β_1-42_ oligomers [60]. TEM images of this protein show the presence of spherical aggregates with diameters of 15–35 nm, while those of the WT c-Src-SH3 domain have a diameter of 20–40 nm. However, in some cases the formation of amyloid oligomers and protofibrils have been described without their conversion to mature amyloid fibers, as for example those of insulin chain B [61]. In view of our results, we cannot totally ascertain whether the c-Src-SH3 fibrils differ entirely or not from the domain-swapped dimers (and the monomer), as has been observed in the case of PI3K-SH3 [62], [63]. Further studies must be performed to clarify this point on the folding pathway of the c-Src-SH3 protein.

When the WT protein was incubated for the same time at 37°C, narrow and elongated amyloid fibrils were obtained. The Gln128Glu mutant shows a different behavior to the WT protein as the formation of amyloid fibrils requires longer periods of time and the addition of salts. After two weeks of incubations, long amyloid fibrils of the Gln128Glu mutant were present in the TEM images (Fig. 8D).

## Discussion

### 3D-Domain swapping in the c-Src-SH3 domain

The results obtained in this work suggest that the partially unfolded c-Src-SH3 domain might fold, under proper conditions, in an intertwined oligomer or a monomer. Both species are present in solution and the addition of different reagents modulates the self-association of c-Src-SH3: low molecular weight PEGs seems to shift the equilibrium to the formation of the dimer; while the addition of high affinity PRMs results in the formation of the PRM-complex of the monomeric form [41]. Besides, as it has been observed in the Eps8 SH3 [18], the equilibrium between the dimeric and monomeric forms of the c-Src-SH3 is also pH-dependent. However, Eps8 crystals of the intertwined dimer were obtained at pH 7.0, and those of the monomer at pH 4.0; comparison of the Eps8 dimeric and monomeric structures lead the authors to conclude that some salt-bridges were responsible of the stabilization of the domain-swapped form [64]. The availability, for the first time, of the monomeric and dimeric crystal structures of the c-Src-SH3 allows us to study the interactions responsible for the stabilization of each form.

The monomeric structures of the c-Src-SH3 (WT and Gln128Glu mutant) have two molecules at the AU. If we compare the same chain between the two c-Src-SH3 structures, there are not significant differences, but if we compare the different chains (A and B) in the same structure, the differences are larger (Table 3). The hydration shell and hydrogen-bond scaffolding around position 128 are different for each chain and these changes are correlated with a displacement of the distal loop position and, at the same time, a different conformation of Leu100, which affects to the hydrogen-bond network around the binding site. Consequently, changes in the Leu100 rotamer would act as a conformational switch to allow the binding of the PRMs in the two available orientations, and the arrangement of hydrogen-bonds around this position might modulate the affinity of the binding.

Comparison of the crystallographic structures of the monomeric (WT_M_ and Q128E_M_) and dimeric (WT_D_ and Q128E/K/R_D_) forms of the c-Src-SH3 domain show that the hydrogen-bond pattern of the dimeric structures resembles that found at chain A of the monomeric ones: (a) residue 128 is oriented to the cavity formed by the distal loop and the diverging β-turn; (b) Leu100 rotamer acquires the same conformation; and, (c) Asp99 and Tyr92 are hydrogen-bonded. In the dimeric structures, Asp99 is modeled in two conformations (S2 Figure): both are linked to Tyr92, but only one is bound to the PEG molecule found in the interface of the intertwined dimer. DLS and NMR experiments indicate that the PEG molecule facilitates the stabilization of the intertwined dimer at lower protein concentrations. Indeed, this molecule enables the formation of several hydrogen-bonds that are not present in the monomeric structure and might energetically favour the formation of the dimeric species [12]. This molecule does not seem to be required for formation of the intertwined dimer, which is formed by itself in solution at high protein concentrations as can be seen in the DLS and NMR experiments. Besides, the crystallographic structures of other SH3 domain-swapped structures do not present any molecule at the interface [34], [64]. However, the presence of the PEG molecule in the structures of the c-Src-SH3 indicates that the formation of this intertwined dimeric conformation can be favoured by small-molecule binding as to become the predominant species in solution. At the same time, we have evidence that the binding of PRM motifs favours the formation of the monomeric form [41]. In this way, the presence of different ligands bound to this SH3 domain might have important implications respect to the biological significance of SH3 oligomerization. A deeper knowledge of how these molecules interfere with the aggregation process might provide significant information for the development of therapeutic approaches to avoid the formation of amyloid aggregates.

Our structures show that the protonation state of some ionizable residues might play a key role in formation of the intertwined dimer. Actually, experiments performed at pH 7.0 indicate that the WT c-Src-SH3 is always a monomer at all protein concentrations assayed in the range 0.14–3.62 mM (1–25 mg·ml^−1^). At this pH, the interaction Asp117_A_-Asp117_B_ is not possible, and we suggest that this interaction could play an important role in the stabilization of the dimeric form. This interaction is also present at the intertwined dimer of a chimeric c-Src-SH3 domain obtained at mild acid pHs [34]. In this structure, the domain swapping takes place concomitantly through two hinge loops, the RT-loop and the n-Src-loop, and no PEG molecule has been found at the dimer interface.

### Amyloid fibril formation

In the c-Src-SH3 domain, the formation of intertwined dimer by the swapping of the RT-loop was predicted by MD calculations by Ding and co-workers. These authors proposed that, in some cases, the formation of an open monomer could yield amyloid fibrils if the swapping among different protomers is not reciprocal but propagational [8]. This kind of assembly into amyloid fibrils has been proposed to be underneath the mechanism suggested for some domain-swapping/amyloid-forming proteins, as for example GB1 [65], [66]. Furthermore, these oligomeric structures deserve attention since in several cases in the development of amyloid diseases the toxicity has been attributed to the oligomeric assemblies of the protein, rather than to the docking of mature fibrils [15], [67], [68].

The formation of amyloid fibrils in SH3 domains has been described previously for two members of the family of Src tyrosine kinase: c-Yes [23] and Fyn [24]. Other two SH3 domains have also been described to form amyloids: the N47A mutant of the α-spectrin [26] and PI3K [25]. Both proteins form amyloids at acid pHs (PI3K at pH 2.0 and α-spectrin at pH 3.0) and moderate temperatures (25–37°C). NMR studies performed with the PI3K SH3 domain indicate the presence of partly folded conformations in the acid-unfolded state [69], but not the presence of domain-swapped forms. In the α-spectrin SH3 domain, the presence of dimers and other higher order oligomers have been described [28], but not domain-swapped forms. An alignment of the sequences of the five forming-amyloid SH3 domains is shown at S1 Figure. This alignment shows the high identity among the sequences of the three members of the Src family of tyrosine kinases, and one of the few different residues among these SH3 domains is found at the position 128 (c-Src-SH3 sequence): glutamine in c-Src-SH3, lysine in Yes-SH3 and glutamate in Fyn-SH3. This position is located at the distal loop, as the amyloidogenic mutation of the α-spectrin.

We have studied the stability of the WT and three different mutants of c-Src-SH3 at the position 128. Interestingly, this single mutation affects the protein stability in a pH-dependent manner that is related to the charges present in the pocket formed by the distal loop and the diverging β-turn. Remarkably, the mutation of glutamine by glutamic acid results in a change of the pH where amyloid fibril formation occurs: Gln128Glu mutant develops amyloid fibrils at neutral pH but not a mild acid pHs. However, when a positively-charged residue is present at position 128 (arginine or lysine), we did not observe amyloid formation; this further indicates a key role of residue at position 128 in the folding and subsequent formation of amyloid aggregates. The mutant position studied at this work is next to an amyloid forming sequence (see S1 Figure) and differences in the charge can affect to the amyloid formation. Changes in charge at the mutated residue close to this aggregation-prone region might act as “gatekeepers” and prevent intermolecular associations that would allow the formation of ordered β-sheets via electrostatic charges as it has been described before for the PI3K SH3 domain [62], [70].

Our results suggest some kind of electrostatic interaction between the residue at position 128 and Glu106, whose alteration might be responsible for the opening of the hinge loop leading to the observed swapped-structures. These residues are not in direct contact, indeed a cluster of water molecules is placed between them, where W1 is the sole molecule present in all the structures; this water molecule is linked to Glu106 side-chain, which is also hydrogen-bonded to Ser123 (chain A and intertwined dimer) or Thr125 (chain B) side-chains. The hydrogen-bond between Glu106-Ser123 has been previously described to play a key role in the folding reaction of the c-Src-SH3 domain, bringing together the distal loop and the diverging β-turn [3]. Nevertheless, we have structural evidence of the formation of a hydrogen-bond between Glu106-Thr125 side-chains in chain B of the monomeric structures (WT_M_ and Q128E_M_). This interaction seems to be correlated with the conformational changes and the water network arrangement described above. Furthermore, we have found an equivalent interaction (Glu107_Fyn_-Thr126_Fyn_) in the structure of an intermediate state of folding of the Fyn-SH3 domain solved by NMR relaxation dispersion spectroscopy [24].

Based on the above findings, we have analyzed the possible amyloid-formation routes. In the WT protein, a first step for the development of c-Src-SH3 oligomeric aggregates, which results in the formation of amyloid fibrils, might be an equilibrium between the monomer and the partially unfolded dimer. In this open-like promoter, the structure around the folding nucleation site, formed by the distal loop and the diverging β-turn, seems to be preserved [34]. For this partial unfolding of the dimer to occur, the n-Src loop must show some flexibility as it requires several torsion angles of its residues to be extensively rotated [12]. Our structures show that the flexibility of the n-Src loop is only apparent in the chain B of the monomeric structures, and it seems to be linked to the conformational changes at the folding nucleation site. This scenario agrees with the MD calculations which suggest that the folding of the c-Src-SH3 is governed by two barriers [71]. The first one, associated with the formation of the main TS for folding, where the interactions between the diverging β-turn and the distal loop are fully structured, while those corresponding to the n-Src loop and RT-loop are loosely formed. The second one is a post-TS desolvation barrier where the release of water molecules joining the hydrophobic sheet takes place. This three-state protein folding pathway has been experimentally proven by NMR relaxation dispersion spectroscopy for several closely related SH3 domains [24], [72]. Within this framework, the intertwined dimer might be the result of the interaction of neighboring SH3 chains in the folding intermediate formed after the major TS [73], where the diverging β-turn and the distal loops are formed, but not the n-Src and the RT-loops [34]. Thus, this intermediate has an alternative fold represented by the intertwined dimer, which is energetically favoured, and where the requirements of the burial of the hydrophobic core of the SH3 domain are satisfied. Nevertheless, in some cases and carried by the high concentration of protein, temperature and/or the addition of other agents, the resulting species is the amyloid.

The participation of intertwined structures as intermediates of amyloid formation has been previously described for several disease-related proteins, as for example cystatin C [19], transthyretin [74] or β_2_-microglobulin [75]. However, it is not clear how the formation of oligomeric domain-swapped structures helps to the amyloid formation and how these aggregates can be toxic [68]. Moreover, solid-state NMR experiments show that, in many cases, the amyloid fibers formed by domain-swapped proteins lack of native structure and adopt the β-arcade amyloid motif (for a review see ref [66]). In fact, the formation of domain-swapped oligomers has not been found in well-characterized amyloid-forming proteins, as it is the case of the PI3K [69] and the α-spectrin SH3 domains [26]. Several NMR studies on the PI3K-SH3 domain show that the backbone conformation of the protein in the fibril form is different from that of the native one [62], [63].

The case of the Gln128Glu mutant amyloid fibril formation is different from the WT protein as it takes place at neutral pH where the protein is monomeric even at high concentrations (>20 mg·ml^−1^). Therefore, an alternative route must be considered where the amyloid aggregation does not require the formation of the domain swapped form. The Gln128Glu mutation emulates the residue present at the position 128 in the Fyn-SH3 domain (Glu129_Fyn_). Fyn-SH3 domain also forms amyloid fibrils at neutral pH [24], but we have neither detected the formation of amyloid at mild acid pH in this domain, nor the formation of oligomeric domain-swapped structures (data not shown). In the Fyn-SH3 domain, and in the c-Src-SH3 Gln128Glu mutant, the presence of two negatively charged glutamate residues in the folding nucleation site would result in the electrostatic repulsion among the residues buried in the hydrophobic pocket. This repulsion might favour the intermediate-like structure in chain B, where the glutamate side chain is oriented outside the pocket formed by the distal loop. The later progress of this intermediate towards the formation of the intertwined dimer is stopped because the interaction between symmetry related Asp117 is not possible at neutral pH, where the aspartic acid is unprotonated. In this way, the opening of the domain and formation of amyloid fibrils at neutral pH occurs slowly, apparently without the participation of an intertwined structure, and it can be accelerated by the presence of high salt concentrations and/or high temperatures.

Other residues might also contribute to the pH-dependent behaviour in the formation of the intertwined dimer and amyloid fibrils. For example, at the diverging β-turn the presence of Lys103 and Lys104 might play some role as they are implied in a cluster of hydrogen-bond/salt-bridge interactions where Asp91, which is adjacent to the C-terminal of β1 strand, is also involved (Fig. 4A). This β-strand belongs to a region of the protein that has been found to fold late in the folding reaction [71]. Calculations performed with the TANGO algorithm [49] indicate that, indeed, the β1-strand and residues adjacent to its C-terminal belong to the most aggregation-prone region of the c-Src-SH3 domain (as it is in the Fyn-SH3 domain [24]). These results are also corroborated by using the WALTZ algorithm [50] (S1 Figure). Recently, Neudecker et al. [24] have solved the structures of the native and a folding intermediate of a mutant of the Fyn-SH3 domain by using NMR relaxation dispersion spectroscopy to study the formation of amyloid fibrils in the Fyn-SH3 domain. We have compared the NMR coordinates of the ensemble with chains A and B of the monomeric structures of the c-Src-SH3 domain. Interestingly, both the hydrogen-bond scaffold and the conformational changes found between chain A and B in the c-Src-SH3 monomeric structure were also observed between the structures of the native (PDB code 2LP5) and intermediate (PDB code 2LP2) of the Fyn-SH3 mutant (S3 Figure). In all the structures of the native form Glu107_Fyn_ side-chain is hydrogen-bonded to Ser124_Fyn_ but not to Thr126_Fyn_ side-chain. In the intermediate structure, two different situations can be found. We have used the web server OLDERADO [76] to determine the cluster of structures and the most representative structure of each one. There are two clusters of structures: the first one (structures 2–4, 6–9), where the structure 2 is the most representative, Glu107_Fyn_ is hydrogen-bonded to Thr126_Fyn_ and Ser124_Fyn_; the second one (structures 1, 5, 10), where structure 5 is the most representative, Glu107_Fyn_ side-chain is hydrogen-bonded to Thr126_Fyn_ but not to Ser124_Fyn_ side-chain. The differences in the hydrogen-bond networks between the native and the intermediate states of the Fyn-SH3 mutant are similar to those found between the two chains present in WT_M_ and Q128E_M_: chain B resembles the structure of the folding intermediate, while chain A is similar to the native one of the Fyn-SH3 domain.

Another important difference between the structures of the folding intermediate and the native state of the Fyn-SH3 domain is the lack of β5-strand in the intermediate structure. The absence of this β-strand results in the loss of the hydrogen bonds between the backbone atoms of the residues belonging to β5 and β1 strands (Tyr137_Fyn_-Leu90_Fyn_ and Ala139_Fyn_ –Glu88_Fyn_). The lack of hydrogen-bonds between β5 and β1 results in the displacement of β1 and the diverging β-turn, which produces changes in hydrogen-bond and salt-bridge interactions around this position. In all these conformational changes, Asp91 seems to play some role. As it has been described above, this residue belongs to the most aggregation-prone region of the c-Src-SH3 domain (S1 Figure), and this cluster of hydrogen-bond and salt-bridge interactions can affect the hydrogen-bond network at the folding nucleation site, which includes different conformers of the residue Leu100. The backbone nitrogen atom of this residue is hydrogen-bonded to the backbone oxygen of Tyr131, which is next to Gly130 (Gly141_Fyn_). NMR studies performed with the Fyn-SH3 Gly130Met and Gly130Val mutants, aimed to determine the presence of folding intermediates, reveal a key role of this glycine residue. This residue is found in ∼95% of the SH3 sequences and shows unusual φ and ψ values [77]. A glycine is not only the most energetically favoured residue at this position by its backbone conformation, also the lack of side-chain allows changes in the position of the rotamers of Leu100.

To sum up, our results show that: (i) electrostatic interactions and solvation of the residues close to the folding nucleation site of the c-Src-SH3 domain might play an important role during the protein folding reaction and amyloid fibril formation; (ii) no amyloid formation has been detected when the position 128 is occupied by a positively charged residue; (iii) amyloid aggregates are formed quickly at mild acid pHs in the case of the neutral residue glutamine placed at that position; and, (iv) the negatively charged residue glutamate results in the formation of the amyloid at neutral pH. All pieces of evidence indicate that the amyloid formation in the c-Src-SH3 domain might proceed via two distinct pathways at mildly acid pH as compared to neutral pH.

## Supporting Information

S1 Figure **Sequence alignment of the amyloid-forming SH3 domains and overall fold.**
(TIF)

S2 Figure **Interactions of the PEG molecule at the interface of the WT intertwined dimer.**
(TIF)

S3 Figure **Comparison of the native and intermediate state structures of the Fyn SH3 domain Ala39Val/Asn53Pro/Val55Leu mutant.**
(TIF)
