# Supplementary figures and images for: Electrostatic Effects in the Folding of the SH3 Domain of the c-Src Tyrosine Kinase: pH-Dependence in 3D-Domain Swapping and Amyloid Formation
Source: PLoS One. 2014 Dec 9;9(12):e113224. doi: 10.1371/journal.pone.0113224 (PMC4260792; doi:10.1371/journal.pone.0113224)

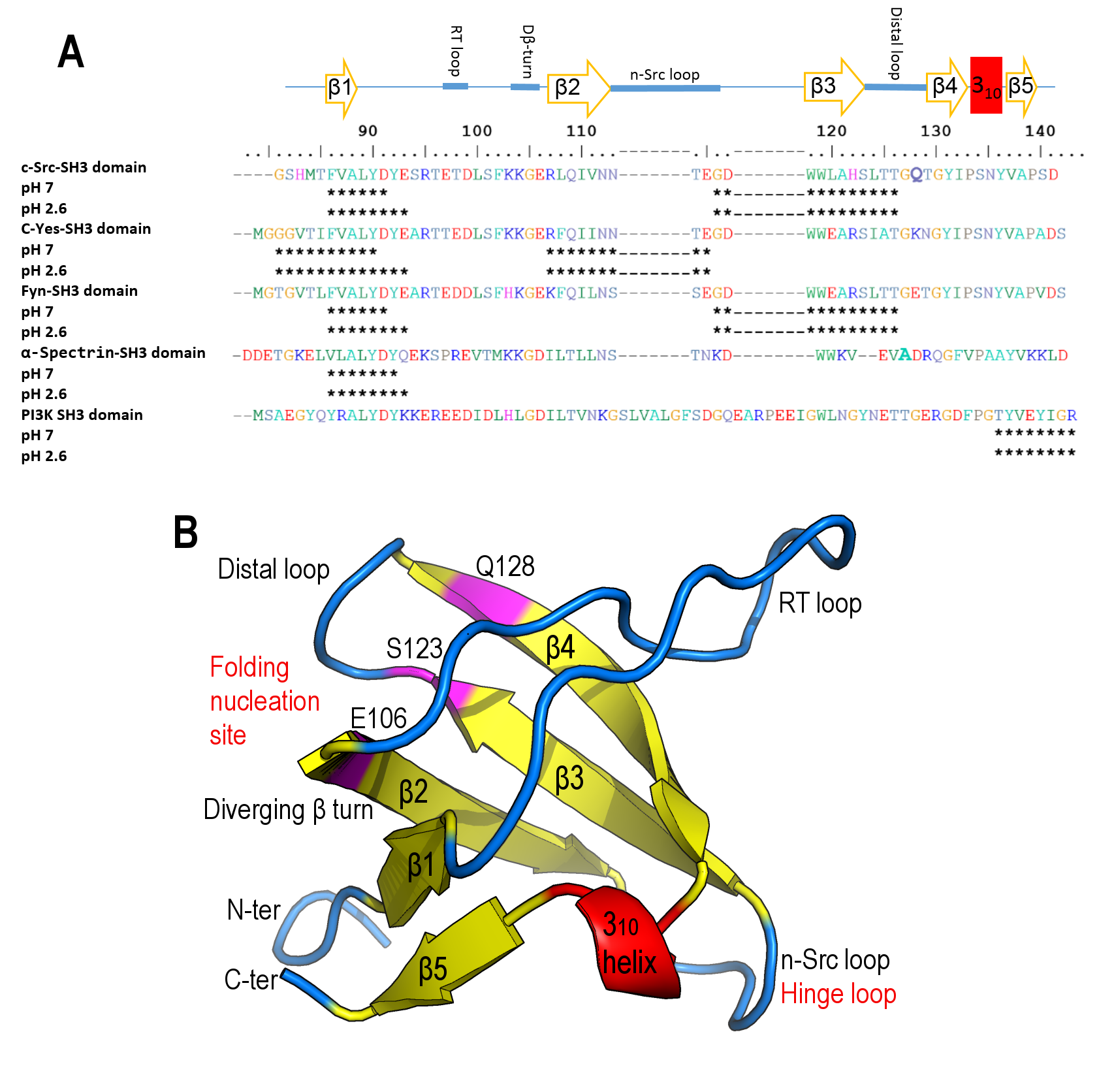

Supplement: S1 Figure — Sequence alignment of the amyloid-forming SH3 domains and overall fold. (TIF) [file pone.0113224.s001.tif]

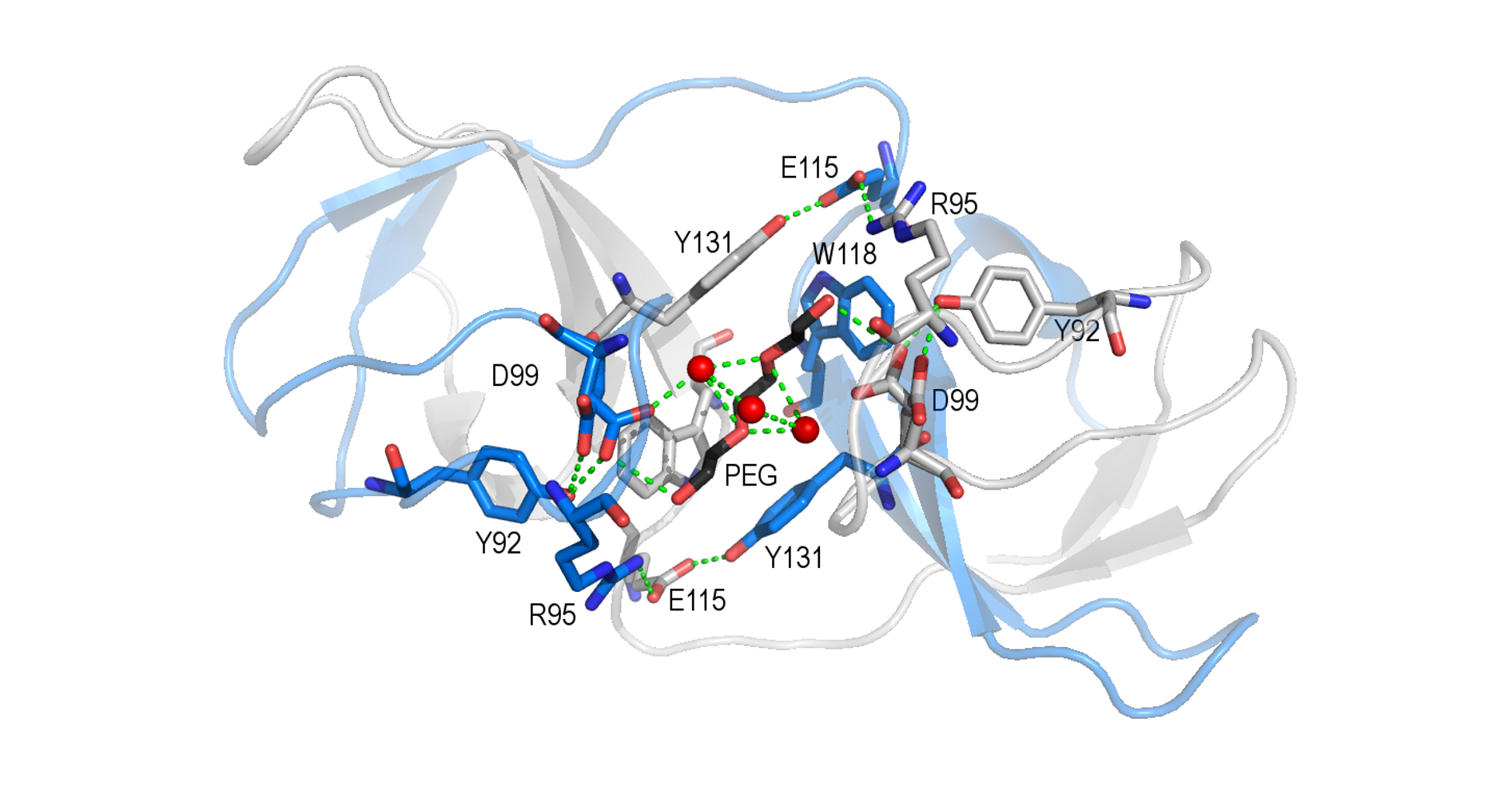

Supplement: S2 Figure — Interactions of the PEG molecule at the interface of the WT intertwined dimer. (TIF) [file pone.0113224.s002.tif]

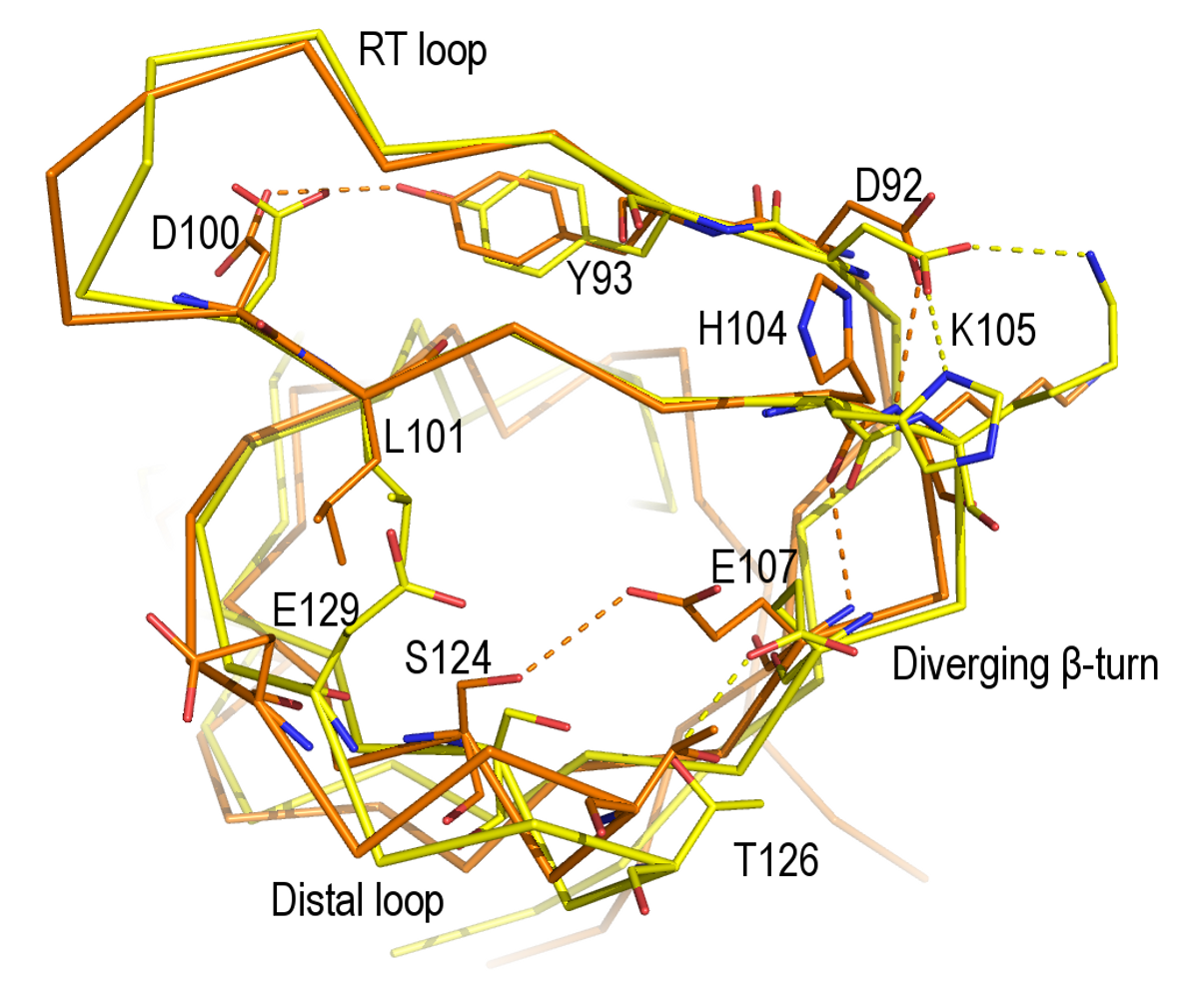

Supplement: S3 Figure — Comparison of the native and intermediate state structures of the Fyn SH3 domain Ala39Val/Asn53Pro/Val55Leu mutant. (TIF) [file pone.0113224.s003.tif]
